# Personalized Nutrient Profiling of Food Patterns: Nestlé’s Nutrition Algorithm Applied to Dietary Intakes from NHANES

**DOI:** 10.3390/nu11020379

**Published:** 2019-02-12

**Authors:** Fabio Mainardi, Adam Drewnowski, Hilary Green

**Affiliations:** 1 Nestlé Research, Vers-chez-les-Blanc, 1000 Lausanne 26, Switzerland; Fabio.Mainardi@rd.nestle.com; 2 Center for Public Health Nutrition, University of Washington, Seattle, WA 98195-3410, USA; adrewnow@fredhutch.org

**Keywords:** nutrient profiling, dietary pattern, nutritional quality, energy density, nutrient density

## Abstract

Nutrient profiling (NP) models have been used to assess the nutritional quality of single foods. NP methodologies can also serve to assess the quality of total food patterns. The objective of this study was to construct a personalized nutrient-based scoring system for diet quality and optimal calories. The new Nestlé Nutrition Algorithm (NNA) is based on age and gender-specific healthy ranges for energy and nutrient intakes over a 24 h period. To promote nutrient balance, energy and nutrient intakes either below or above pre-defined healthy ranges are assigned lower diet quality scores. NNA-generated diet quality scores for female 2007–2014 National Health and Nutrition Examination Survey (NHANES) participants were compared to their Healthy Eating Index (HEI) 2010 scores. Comparisons involved correlations, joint contingency tables, and Bland Altman plots. The NNA approach showed good correlations with the HEI 2010 scores. NNA mean scores for 7 days of two exemplary menu plans (MyPlate and DASH) were 0.88 ± 0.05 (SD) and 0.91 ± 0.02 (SD), respectively. By contrast, diets of NHANES participants scored 0.45 ± 0.14 (SD) and 0.48 ± 0.14 on first and second days, respectively. The NNA successfully captured the high quality of MyPlate and Dietary Approaches to Stop Hypertension (DASH) menu plans and the lower quality of diets actually consumed in the US.

## 1. Introduction

Nutrient profiling (NP) models were developed to assess nutrient density of individual foods [1] expressed per 100 kcal, 100 g, or per serving [2]. Favorable nutrient profiles have provided the scientific basis for the adjudication of nutrition and health claims and for front-of-pack labels and logos [3]. Unfavorable nutrient profiles, largely linked to excessive food content of calories, fat, sugar, and salt, have been used to develop warning labels and to limit marketing and advertising to children [4,5,6,7]. The food industry has also used NP modeling methods to evaluate and (re)formulate product portfolios [8]. 

While most of the existing NP models apply to single foods, the current emphasis in public health nutrition is on nutrient density of habitual food patterns [9,10]. NP methods, often based on nutrients-to-calories ratio, were recently used to evaluate the nutrient balance of MyPlate meals [11]. Most recently, the Nutrient Rich Food (NRF9.3) index was used for a standardized analysis of diet quality for children and adults in nationally representative nutrition surveys from Canada, Denmark, France, Spain, UK, and the US [12,13,14,15,16,17,18]. NP modeling was used to assess diet quality across countries in preference to alternative diet quality measures, such as the Healthy Eating Index (HEI).

Initially developed in 1995 [19], the HEI is a 100 point scale that measures compliance with the Dietary Guidelines for Americans, which are revised and reissued every 5 years [20]. Following the Dietary Guidelines, the HEI incorporates concepts of adequacy and moderation. The most recent versions have incorporated food groups to encourage (e.g., leafy green vegetables, whole fruit), food groups to limit (refined grains) as well as desirable nutrients (plant protein) and desirable nutrient ratios (saturated to unsaturated fat). Adjusting the HEI for 1000 kcal means that diet quality scores do not increase with higher energy intakes.

Given that the HEI is a hybrid tool, inclusive of both nutrients and food groups, its calculations critically depend on the availability of the Food Patterns Equivalents Database (FPED) formerly known as the MyPyramid Equivalents Database (MPED). The FPED converts the foods and beverages in the Food and Nutrient Database for Dietary Studies to the 37 United States Department of Agriculture (USDA) Food Patterns components that are used to calculate HEI. While FPED data are available from the USDA, they have not been calculated by agencies in Canada, Denmark, France, Spain, or the UK. For that reason, the HEI measure cannot be used outside the US. 

Some countries have developed similar indices to monitor compliance with local dietary guidelines. Examples include Canada [21], Spain [22], Brazil [23], and Australia [24]. However, those approaches are far from standardized. In all cases, better compliance with dietary guidelines—variously assessed through food or nutrient consumption thresholds, ranges, or daily amounts—led to a higher diet quality scores. 

The NNA is a new nutrient-based model that assesses the nutrient density of dietary patterns with no need of MPED or FPED databases. As such, the present NNA model can be applied worldwide, wherever population energy and nutrient intake data are available. Unlike the HEI approach, the NNA model is energy adjusted. Therefore, in contrast to the HEI model, under- or over-consumption of energy and nutrients leads to lower scores. This paper provides the scoring system, together with the steps taken to demonstrate its validity and reliability. 

## 2. Materials and Methods 

### 2.1. Nestlé Nutrition Algorithm (NNA)

**Figure 1:**
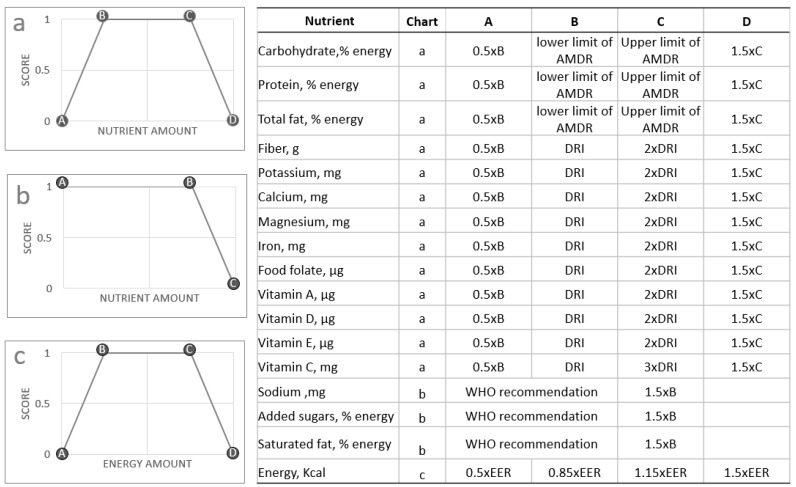
The Nestlé Nutrition Algorithm scoring system. AMDR: Acceptable Macronutrient Distribution Range, DRI: Dietary Reference Intakes, WHO: World Health Organization, EER: Estimated Energy Requirement.

The new Nestlé’ Nutrition Algorithm approach was to award maximum scores to consumption patterns that kept both energy and nutrients within the healthy range. The NNA score, illustrated in Figure 1, was based on three components. 

Consumption patterns within the healthy range received a score of 100. 

Consumption patterns below the healthy range received a diminishing score from 100 to 0.

Consumption patterns above the healthy range received a diminishing score from 100 to 0. 

The NNA score (a number from 0–100) for dietary nutrient quality was derived from the average of nutrients included in the model for a given period of time. The model was not weighted but could be weighted in the future (or not). The period of time was 24 h but that could be different in the future (or not). This nutrient score was then multiplied by the energy score, so that intakes outside the predefined healthy energy range received lower scores. 

The scoring system is shown in Figure 1. Chart a illustrates the way that points are awarded for carbohydrate, protein, total fat, fiber, potassium, calcium, magnesium, iron, food folate, and vitamins A, D, E and C. If the nutrient amount falls between Point B and C then it would receive a maximal score of 1.0. Nutrient amounts that fall between A and B, or between C and D receive partial scores. Nutrient amounts at or less than point A, or at or greater than point D receive a score of zero. The values used to define these 4 points (A, B, C and D) are provided in the adjacent table in Figure 1.

Chart b illustrates the way that points are awarded for sodium, added sugars and saturated fat. If the nutrient amount falls between Point A and B then it would receive a maximal score of 1.0. Nutrient amounts that fall between B and C receive partial scores. Nutrient amounts at or greater than point C receive a score of zero. The values used to define these 3 points (A, B and C) are provided in the adjacent table in Figure 1.

Chart c illustrates the way that points are awarded for energy. If the energy amount falls between Point B and C then it would receive a maximal score of 1.0. Energy amounts that fall between A and B, or between C and D receive partial scores. Energy amounts at or less than point A, or at or greater than point D receive a score of zero. The values used to define these 4 points (A, B, C, and D) are provided in the adjacent table in Figure 1.

#### 2.1.1. The Selection of Index Nutrients

The present calculations were based on the nutrient values for foods in the USDA’s National Nutrient Database for Standard Reference (SR), release 28. Nutrients that were selected for inclusion in the NNA were carbohydrate, protein, total fat, fiber, potassium, calcium, magnesium, iron, food folate, vitamin A, vitamin D, vitamin E, vitamin C, sodium, added sugars and saturated fat. These nutrients had been identified to be either shortfall nutrients, or nutrients consumed in excess in the US diet [25]. Values for added sugar were extracted from the USDA’s Food Patterns Equivalents Database (2011–2012).

#### 2.1.2. Defining Healthy Ranges for Nutrients

The healthy ranges for each nutrient are based on age and gender specific Dietary Reference Intakes (DRI) (i.e., Recommended Dietary Allowance where available or else Adequate Intakes). For most micronutrients, we define the healthy range as 100–200% DRI, except vitamin C for which we define the healthy range as 100–300%. For sodium, saturated fat and added sugars the healthy range was defined as 0–100% of levels recommended by the World Health Organization. The healthy range for macronutrients was defined using the Acceptable Macronutrient Distribution Ranges (AMDRs) recommended by the Food and Nutrition Board, Institute of Medicine, National Academies. For insufficient micronutrient intakes, a score of zero was given when intake was ≤(0.5 × DRI). For micronutrient intakes above the healthy range, a score of zero was given when intake was ≥(1.5 × the upper healthy range). The present upper limit (200% DRI) is distinct from, and much lower than, the Tolerable Upper Limit (TUL) established for some nutrients by regulatory authorities and expert panels. However, if personalization of the algorithm generates a value for point D (Figure 1) that is greater than the TUL then point D should be the TUL (and not 1.5 × C). 

#### 2.1.3. Defining Healthy Ranges for Energy

Age- and gender-specific estimated energy requirements (EER) were based on equations provided by the Institutes of Medicine (2002) [26]. The healthy range for energy was based on 15% deviations below or above the calculated value. For implausible energy intakes, a score of zero was given when total energy intake was ≤(0.5 × EER). For excessive energy intakes, a score of zero was given when total energy intake was ≥(1.5 × EER).

### 2.2. NNA Applied to MyPlate and DASH Menu Plans 

The selection of upper healthy ranges for nutrients of interest was compared to data from two publicly available menu plans that represent healthy food patterns: MyPlate [27], created by the USDA and DASH [28], sponsored by sponsored by the National Institutes of Health. Each menu plan provided complete meals (breakfast, lunch and dinner), snacks and beverages for 7 consecutive days. The nutrient composition of each menu plan was derived and reported in Appendix A. The menu plans are provided in Appendix B. The NNA scores were calculated for each of these menu plans.

Mean energy and nutrient content of the food patterns were converted to percent DRIs in order to see if they fall within the healthy ranges defined in Figure 1. 

### 2.3. NNA Applied to NHANES 2007–2014 Dietary Intakes

NHANES data (2007–2014) were used to test the validity of the algorithm. The nutrient intakes of subjects with different profiles were scored with the NNA. For simplicity, some of the results below are illustrated only for non-pregnant women aged 31–50 years, assuming an energy requirement of 2000 kcal. Section 3.3.4. below compares the NNA scores for different sub-populations, stratified by age, gender or socio-economic status.

### 2.4. Statistical Analysis

Table 1 summarizes the steps taken to assess the validity of the NNA.

The statistical relationship between the total score and the single nutrient scores was evaluated through principal component analysis (PCA) and Cronbach’s coefficient. PCA was used to assess the “true” underlying dimensionality of the data, while Cronbach’s coefficient provided a measure of internal consistency, as a function of the number of items, the average covariance between item-pairs, and the variance of the total score. 

#### 2.4.1. Comparisons between NNA and HEI-2010 Using NHANES 2011–2012

The Healthy Eating Index (HEI) is a widely used measure of dietary quality. It was designed to assess diet quality and effectively it assesses the extent to which the US population adheres to the Dietary Guidelines for Americans. Although the HEI was constructed differently from our nutrition algorithm, we hypothesize that there should be agreement between these two scoring mechanisms. Therefore, we assessed the performance of our nutrition algorithm against HEI 2010, for NHANES 2011–2012. 

We illustrate this for the sub-population of females 31–50 years. We scored the NHANES 2011–2012 data for day 1 with the 2010 version of HEI, and compared the results with the present NNA. To do this the simple HEI scoring algorithm method was used [29]. Nutrient analyses were based on the USDA’s Food and Nutrient Database for Dietary Studies (2011–2012) and the Food Pattern Equivalents Database (2011–2012). 

The data were stratified by age and gender to yield four subgroups: male 31–50 years, female 31–50 years, male 70+ years, and female 70+. The resulting NNA scores were compared pairwise, with the T-test (null hypothesis: mean scores are equal). 

NHANES participants were also assigned to a socio-economic category, ”low”, “medium” or “high”, following the same methodology as in Wang et al. (2014) [30]. The mean NNA scores were compared between the three groups, and differences between groups were assessed using a Kruskal–Wallis test for comparisons.

#### 2.4.2. Internal Consistency

We assessed internal consistency, using the Cronbach’s coefficient. This statistic evaluates whether the different components in the score are really measuring the same construct. The Cronbach coefficient can range from 0 to 1, a higher score indicating a higher internal consistency. As a rule of thumb, values above 0.7 are generally considered to be acceptable. 

### 2.5. NNA Applied to FPED Food Groups

In order to test whether NNA is associated with certain food patterns, we calculated the intakes of some specific food groups using the Food Patterns Equivalent Database, and compared their distributions between NNA tertiles. The Food Patterns Equivalents Database (FPED) converts the foods and beverages in the Food and Nutrient Database for Dietary Studies to the 37 USDA Food Patterns components, and it is publicly available. We used FPED 2011–2012 to calculate the intakes of the following food groups in a subset of the NHANES participants: (a) dark green vegetables; (b) red and orange vegetables (excluding tomatoes); (c) cured meat; (d) citrus, melons, and berries; (e) solid fats; (f) whole grains; (g) refined grains; and (h) whole fruits. We refer to the FPED documentation for the exact definition of which foods are included in each group [31].

We considered non-pregnant women aged 31–50 years, energy intake of 1700–2300 kcal. We split the dataset in NNA tertiles and compared the distributions of intakes for each food group.

## 3. Results

### 3.1. NNA Applied to MyPlate and DASH Menu Plans 

The MyPlate and DASH menu plans provided 7 days of complete meals (breakfast, lunch, and dinner), snacks and beverages. The NNA scores for the 7 days of each of these menu plans were. 0.88 ± 0.05 (SD) for MyPlate and 0.91 ± 0.02 (SD) for DASH. 

#### Relative Validation of Healthy Ranges

**Figure 2:**
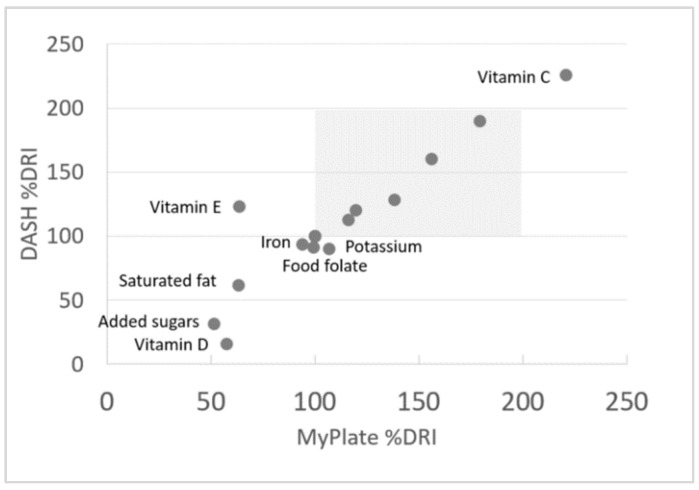
Dietary Reference Intakes (DRIs) for nutrients values from MyPlate and DASH menu plans for non-pregnant women aged 31–50 years. This shows the relationship between the nutrient composition of the DASH menu plan and the MyPlate menu plan, expressed as percent of the dietary reference intake for each nutrient. Each data point represents the mean of 7 days of each menu plan (the menu plans are provided in Appendix B). DASH: Dietary Approaches to Stop Hypertension.

A scatterplot of percent RDIs for the mean nutrient values from the MyPlate and DASH menu plans are shown in Figure 2. Figure 2 shows that vitamins and minerals in MyPlate and Dash meals were mostly within the 100%–175% daily value range. Given the emphasis on vegetables and fruit in MyPlate and in DASH, vitamin C levels were far in excess of requirements. Both MyPlate and DASH were careful to limit saturated fat, added sugars, and sodium—mean values were at or below maximum recommended values. Vitamin E was short of the DRI in MyPlate (not in DASH) whereas vitamin D was low in both. 

The present choice of 100%–200% of age and gender specific RDAs was thus validated relative to MyPlate and DASH.

### 3.2. NNA Applied to NHANES 2007–2014 Dietary Intakes

The NNA scores for the non-pregnant women aged 31–50 years who took part in the NHANES dietary surveys between 2007–2014 were 0.45 ± 0.14 (SD) and 0.48 ± 0.14 (SD) for days 1 and 2, respectively. Sample sizes were 743 and 605, respectively. The scores for individual nutrients are shown in Table 2. 

### 3.3. Comparisons between NNA and HEI-2010 using NHANES 2011–2012 

For simplicity, since the definition of the Healthy Eating Index has changed over time, we limit the comparison between NNA and HEI to the HEI 2010 and the 2011–2012 cycle of NHANES.

#### 3.3.1. Correlations

**Figure 3:**
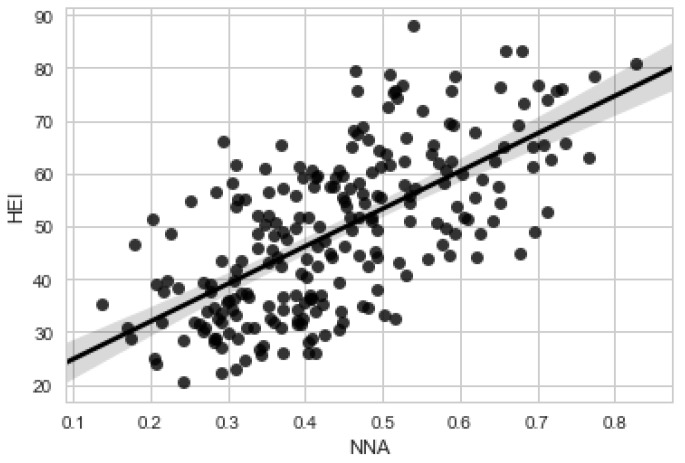
Scatterplot, NHANES 2011–2012, females 31–50 years, energy intake between 1700 and 2300 kcals, day 1 (*n* = 155). This shows the relationship between the NNA score (x axis) and the HEI score (y axis) for a subset of NHANES data. NHANES: National Health and Nutrition Examination Survey, NNA: Nestlé Nutrition Algorithm, HEI: Healthy Eating Index.

We first calculated the Pearson correlation coefficient between the two scores, without any restrictions on energy intake (*n* = 1348 women, 31–50 years). Then we restricted the analysis to those women whose caloric intakes were between 1700 and 2300 kcal (Day 1, *n* = 155; Day 2, *n* = 135). The data are shown in Figure 3.

Pearson correlation coefficients between HEI and the NNA for women aged 31–50 years and without energy restriction were 0.32 for Day 1 and 0.22 for Day 2. With energy restriction, the correlations were 0.69 (Day 1) and 0.54 (Day 2).

#### 3.3.2. Bland–Altman Plots (Women Aged 31–50 Years, Non-Pregnant and Non-Lactating)

**Figure 4:**
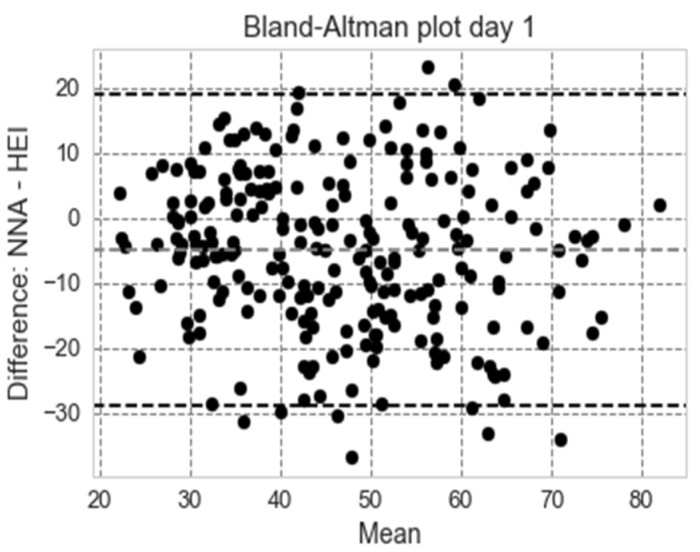
Bland–Altman plot showing agreement between HEI and the NNA score for women aged 31–50 years non-pregnant and non-lactating (NHANES 2011–2012, day 1, *n* = 155). NHANES: National Health and Nutrition Examination Survey, NNA: Nestlé Nutrition Algorithm, HEI: Healthy Eating Index.

A standard way to evaluate the agreement between two methods of measurement is through Bland–Altman plots [32]. The Bland–Altman analysis shows a bias of −4.48 (day 1) and −3.84 (day 2), meaning that our nutrition score is generally lower that HEI 2010; in addition, 95.04 percentage points (day 1) and 95.62 percentage points (day 2) were within the limits of agreement. The Bland–Altman plots for agreement (with 95% confidence), restricted to the 1700–2300 range, are shown in Figure 4 for day 1.

#### 3.3.3. Analysis by Quartile (Women Aged 31–50 Years, Non-Pregnant and Non-Lactating)

**Figure 5:**
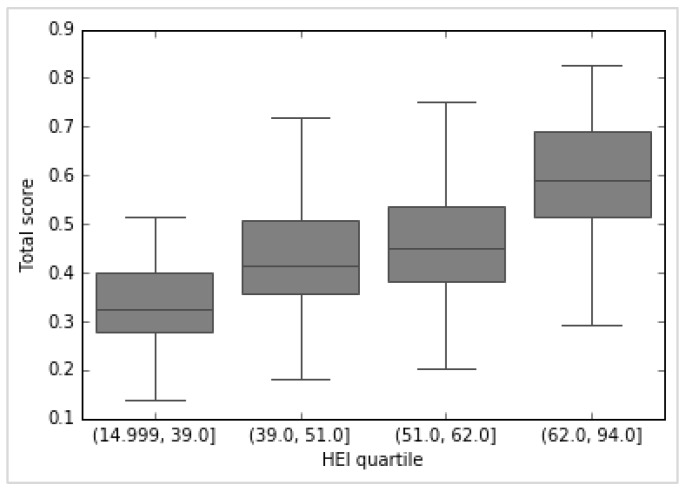
HEI vs. NNA score by quartile for day 1 for women aged 31–50 years, non-pregnant and non-lactating (*n*= 155). This shows the relationship between the HEI score by quartile (x axis) and the NNA score (y axis) for a subset of NHANES data. NHANES: National Health and Nutrition Examination Survey, NNA: Nestlé Nutrition Algorithm, HEI: Healthy Eating Index.

As a further means of comparison, the NHANES dataset (again with calories restricted to 1700–2300) was split by HEI quartiles and the mean nutrition algorithm scores were compared between the groups. This is shown in Figure 5 below for day 1 (the data for day 2 are virtually the same). The Tukey test rejected the null hypothesis of equal means (95% confidence level) for both days.

#### 3.3.4. Impact of Age, Gender, and Socioeconomic Status

A comparison between NNA and HEI scores according to age, gender, and socioeconomic status is provided in Table 3 and Table 4. 

Socio-economic status (SES) was defined as in [30]: categorization of socioeconomic status (SES) was based on education and income level. Income level was categorized according to the poverty income ratio (PIR) as: (a) less than 1.30; (b) 1.30 to 3.49; and (c) 3.50 or higher. Years of formal education were categorized as: (a) less than 12 years; (b) completed 12 years; (c) some college; and (d) completed college. Participants with more than 12 completed years of educational attainment and a PIR of at least 3.5 were categorized into high SES; participants with less than 12 years of educational attainment and a PIR less than 1.30 were categorized into low SES; and others were classified as medium SES.

### 3.4. Validation against Food Groups

Table 5 shows the average intakes in each NNA tertile, as well as the *p*-value of a Kruskal–Wallis test of comparison (the null hypothesis being that there is no difference between the distributions). All *p*-values are less than 5%, except in the case of refined grains. 

### 3.5. Internal Consistency

The results for the Cronbach’s alpha coefficient are summarized in Table 6 using a subset of data from NHANES 2011–2012: Female 31–50 years, 1700–2300 kcal.

### 3.6. Principal Component Analysis

**Figure 6:**
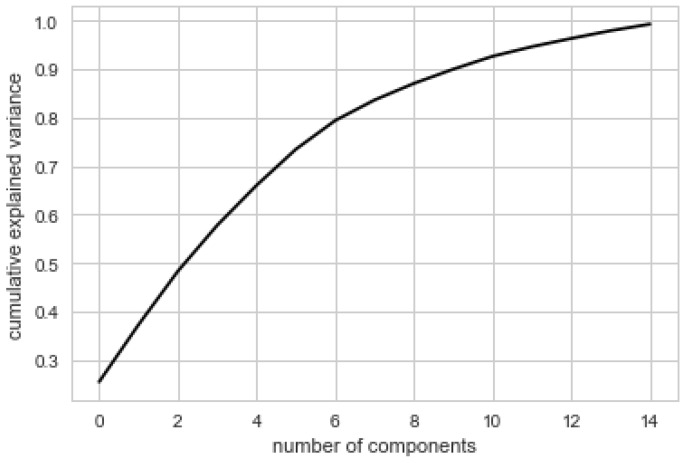
PCA analysis: proportion of variance explained by the principal components of the algorithm. Data = NHANES 2011–2012, day 1, women aged 31–50 years, non-lactating or pregnant (*n* = 155). This shows the amount of variance between each of the nutrients in the model. PCA: principal component analysis.

Figure 6 below shows the proportion of variance explained by the principal components. Fifteen principal components were selected by maximum likelihood estimation [33]. This suggests that the dimensionality cannot be substantially reduced. Principal component analysis confirmed that a number of components (nutrients) independently contribute to the overall score. In other words, the PCA provides evidence that no one single linear combination of the components of the NNA accounts for a substantial proportion of the covariation in dietary patterns. In order to explain at least 90% of the variance, one needs at least nine or 10 factors. It should be noted that the principal components are linear combinations of nutrient scores, and not just nutrient scores. 

## 4. Discussion

The data analysis presented in this paper supports the reliability and validity of the new Nestlé Nutrition Algorithm. Exemplary menu plans are a useful way of testing the construct validity of a diet score, and has been used previously with the HEI [34]. The NNA algorithm could differentiate between the nutritional quality of two exemplary menu plans (as proxies for healthy diets) and the nutritional quality of NHANES participants (with similar energy intakes). The score is derived from 16 nutrients, as well as energy, meaning that no single nutrient influences the overall score. Rather, it is the overall nutrient signature that influences the score, with maximal scores being obtained for exemplary menu plans. Although the scoring system is based on nutrients, it can predict all of the food groups that we looked at, except for refined grains. This means that low NNA scores can be improved by increasing the diversity of food groups.

The internal consistency of the nutrition algorithm is good, as indicated by a Cronbach’s Alpha score of 0.73 using NHANES data. However, the correlation matrix indicates that there are associations between numbers of nutrients. The associations between potassium, magnesium, folate, and fiber might be explained by the coexistence of these nutrients in vegetables. The associations between vitamin A, vitamin D, and calcium could be explained by the coexistence of these nutrients in dairy products. 

This paper has shown that higher scores are obtained for the diets of women of middle and high socio-economic status versus women of low socio-economic status. This is expected, as it is consistent with previous findings of a positive association between indices of socio-economic status and micronutrient intake and status [35].

The NNA approach is to award maximum scores when both energy and nutrients fall within an ideal healthy range. Lower scores are given when nutrients are either above or below this range. This approach differs from other dietary scoring methods in which a maximal micronutrient score is capped at 100% of the nutrient requirements [3,11]. Nevertheless, the idea of an optimal range for nutrients is not new. Twenty years ago, Wirsam and Uthus (1996) proposed a new mathematical approach for scoring the nutritional quality of diets based on fuzzy logic [36]. They assigned values to the intake of nutrients, with values increasing from zero to a maximum of 1.0 when the optimal level was reached, and thereafter falling when the amount exceeded the optimal level and became harmful to health. By creating and combining sets of scores for numerous nutrients, the authors could demonstrate how closely a diet met the national recommendations [36]. Their model included all nutrients (i.e., they were not specifically selected) and there was no consideration of energy. This approach has more recently been applied to food groups for a range of energy levels as means of developing healthy diets [37]. 

An important point of differentiation between the present algorithm, and others, is the energy adjustment. This novel adjustment means that when a 24 h diet is within 15% of energy needs, the score reflects only dietary quality. When energy intake falls outside this ideal range, the score starts to fall, reaching zero when implausibly low or excessive energy levels are reached. NNA scores are not correlated with HEI 2010 scores because HEI measures diet quality independently of quantity. However, when the analysis of NHANES data was restricted caloric intakes between 1700 and 2300 kcal then we observed statistically significant correlations between the HEI 2010 and NNA. The more energy intake is outside the healthy range, the greater is the negative impact of energy on the overall score. The goal is to encourage the consumption of nutrient dense foods and optimize calories, consistent with food-based dietary guidelines.

Consequently, it could be interesting to explore the potential for the NNA to serve as a tool for tracking diet quality and quantity at the population level. Tracking dietary quality over time is problematic using the HEI because the algorithm is regularly modified in line with updates to the US dietary guidelines. An advantage of the NNA is that it effectively distinguishes a healthy eating pattern from an unhealthy one, and could therefore be used in other countries with similar nutrient requirements, even though the foods and beverages consumed may be different. The NNA could potentially also be used by individuals interested in tracking their own diet scores. Such an application could include using portable devices (phone, laptop). 

A potential limitation for the use of the algorithm is the availability of nutrient information. Nutrient information is available for a wide range of foods and beverages in relevant nutrient databases, but inevitably, there are many foods and beverages that are not documented in these databases. The nutrition label of packaged foods provides many of the nutrients required, but not all. Added sugars can be a particular problem, since values are not readily available in public databases. However, the introduction of newly-revised labelling information in the US will mean that added sugars as well as vitamin D will be included on the label, although potassium and vitamins A and C will no longer appear. At the same time, databases are increasingly reporting added sugars (e.g., the Australian nutrient database). This has been facilitated by the introduction of robust algorithms for estimating added sugars [38].

An additional limitation of this, or any other diet score, is the quality of food intake data. The measurement of food intake is thwarted by well-known methodological problems such as underreporting, and variability in food intake on different days of the week. Theoretically, dietary recalls, or analyzing the nutrient content of a duplicate diet, would be the most reliable ways of generating food intake data [39]. The advent of new technologies that can be used with hand held devices such as Smartphones, will help individuals to capture food intake in real-time more reliably. Such technologies include the use of photographs, [40] barcodes, and voice [41]. 

## 5. Conclusions

In conclusion, the NNA provides a reliable and valid method of scoring the healthiness of diets, based on healthy ranges for nutrient composition and energy. It is designed for evaluating the healthiness of the diets of males and females of all ages and diverse energy requirements.

## 6. Patents

Fabio Mainardi and Hilary Green (WO2018234083) System and methods for calculating, displaying, modifying, and using single dietary intake score reflective of optimal quantity and quality of consumables. US first filing of patent, 23 June 2017; Published 27 December 2018. 

## Figures and Tables

**Table 1:** Strategies used to validate the new Nestlé Nutrition Algorithm (NNA) (NHANES: National Health and Nutrition Examination Survey, HEI: Healthy eating Index).

Question	Strategy
Content validity ● Does the score capture the key aspects of diet quality as specified in the current Dietary Guidelines for Americans?	● Compare the NNA scores against the key recommendations
Construct validity ● Does the score give high ranking to menus developed by nutrition experts?	● Compute scores for sample menus generated according to the Dietary Guidelines for Americans
● What is the underlying structure of the score?	● Principal component analysis
● Does the score distinguish between groups with known differences in diet quality?	● Compare scores for different groups in NHANES data
● Does the score agree, to a reasonable extent, with already existing trusted dietary indices?	● Comparison with HEI 2010 on NHANES data,
Reliability ● How internally consistent is the total score?	● Calculate Cronbach coefficient

**Table 2:** NNA scores (individual nutrients and total score) for non-pregnant women aged 31–50 years, energy intake of 1700–2300 kcal (*n* = 1348).

	Day 1 Scores		Day 2 Scores	
	Mean	SD	Mean	SD
Added sugars	0.23	0.39	0.28	0.41
Calcium	0.59	0.40	0.62	0.39
Carbohydrate	0.87	0.23	0.87	0.22
Fat, saturated	0.80	0.27	0.81	0.26
Fat, total	0.72	0.37	0.76	0.35
Fiber	0.32	0.37	0.39	0.39
Food Folate	0.18	0.29	0.20	0.31
Iron	0.43	0.34	0.47	0.36
Magnesium	0.61	0.36	0.67	0.34
Potassium	0.16	0.24	0.20	0.24
Protein	0.96	0.14	0.98	0.11
Sodium	0.44	0.33	0.42	0.33
Vitamin A	0.23	0.42	0.30	0.46
Vitamin C	0.43	0.46	0.48	0.46
Vitamin D	0.06	0.18	0.08	0.22
Vitamin E	0.16	0.29	0.16	0.29
Total score	0.45	0.14	0.48	0.14

SD: standard deviation.

**Table 3:** Comparison between NNA and NHANES 2011–2012 (day 1) for age, gender, and socioeconomic status (SES), with energy intakes between 1700 and 2300 kcal.

	HEI 2010	NNA	Sample Size	Standard Deviation HEI	Standard Deviation NNA
Age					
31–50	50.2	45	411	14.2	14.2
70+	56.3	48.7	171	14.4	13
*p*-value	9.54 × 10^−6^	2.13 × 10^−3^			
Gender					
Male	52.9	46.2	274	14.9	13.2
Female	51.2	45.9	308	15.8	14
*p*-value	1.66 × 10^−1^	7.68 × 10^−1^			
SES					
low	48.2	45.1	69	16.6	14.1
medium	50.8	45.1	298	15.5	13.8
high	54.9	48.1	170	14.3	13
*p*-value	1.25 × 10^−3^	5.36 × 10^−2^			

NNA: Nestlé Nutrition Algorithm, HEI: Healthy Eating Index.

**Table 4:** Comparison between NNA and NHANES 2011–2012 (day 1) for age, gender, and socioeconomic status (SES), with no restriction on energy intakes.

	HEI 2010	NNA	Sample Size	Standard Error HEI	Standard Error NNA
Age					
31–50	49.8	23.3	1557	0.4	0.5
70+	55.8	27	649	0.6	0.8
*p*-value	3.77 × 10^−18^	1.18 × 10^−4^			
Gender					
Male	51.1	22.9	1082	0.4	0.6
Female	52.1	25.8	1124	0.5	0.6
*p*-value	8.7522 × 10^−2^	8.75 × 10^−4^			
SES					
low	47.9	20.9	304	0.8	1.2
medium	50.5	23.6	1148	0.4	0.6
high	55.3	27.8	583	0.6	0.9
*p*-value	7.58 × 10^−14^	2.39 × 10^−6^			

NNA: Nestlé Nutrition Algorithm, HEI: Healthy Eating Index.

**Table 5:** Average intakes of selected food groups, split by NNA tertiles for non-pregnant women aged 31–50 years, energy intake of 1700–2300 kcal.

	NNA Tertile		(0, 0.34)	(0.34, 0.47)	(0.47, 0.77)	*p*-Value (Kruskal Test)
Food Group						
**Dark green veg**		cup-eq/1000 kcal	0.072	0.066	0.248	0.000000
**Red orange veg**		cup-eq/1000 kcal	0.036	0.023	0.096	0.000014
**Cured meat**		oz-eq/1000 kcal	0.486	0.3	0.33	0.008600
**Citrus melon berries**		cup-eq/1000 kcal	0.05	0.082	0.169	0.000010
**Solid fats**		g/1000 kcal	18.157	16.496	12.783	0.000006
**Whole grain**		cup-eq/1000 kcal	0.203	0.296	0.674	0.000000
**Refined grains**		cup-eq/1000 kcal	2.491	2.834	2.669	0.170000
**Whole fruit**		cup-eq/1000 kcal	0.203	0.236	0.517	0.000000

NNA: Nestlé Nutrition Algorithm.

**Table 6:** Cronbach’s alpha coefficient of reliability (or consistency). This shows that the internal consistency of the NNA is high, indicating that it and its components provide a reliable diet score.

	Cronbach’s Alpha	Confidence Interval (95%)	Sample Size
**Day 1**	0.85	(0.85, 0.86)	155
**Day 2**	0.87	(0.86, 0.87)	135

## Appendix A

**Table A1:** Nutrient composition of MyPlate and DASH menu plans.

	MyPlate			DASH	
Nutrients	Mean	SD		Mean	SD
Energy, Kcal	2094	212		2286	110
Carbohydrate, % energy intake	54	7		54	2
Protein, % energy intake	19	3		19	2
Total fat, % energy intake	29	7		30	1
Saturated fat, % energy intake	7	2		7	1
Added sugars, % energy intake	5	3		4	2
Calcium, mg	1449	271		1468	202
Food folate, mcg	448	173		412	110
Fiber, g	31	3		34	3
Iron, mg	18	5		19	5
Magnesium, mg	507	64		569	53
Potassium, mg	4891	494		4918	244
Sodium, mg	1825	341		1935	255
Vitamin A, mcg	1127	535		1301	757
Vitamin C, mg	173	98		194	67
Vitamin D, mcg	9	8		3	5
Vitamin E, mcg	10	4		21	2

DASH: Dietary Approaches to Stop Hypertension.

## Appendix B

**Table A2:** MyPlate and DASH menu plans [18,19].

MyPlate 7-Day 2000 Calorie Menus		
Day	Ingredients	Measure (g)
	Day 1	
Breakfast	Uncooked oatmeal	41
	Fat-free milk	245
	Raisins	18
	Brown Sugar	25
	Orange Juice (unsweetened)	248
Lunch	Tortilla chips	57
	Cooked ground turkey	57
	Corn & Canola oil	9
	Kidney beans	46
	Low-fat cheddar cheese	14
	Chopped lettuce	18
	Avocado	115
	Lime juice	6
	Salsa	36
	Coffee	179
Dinner	Lasagna noodles	57
	Cooked spinach	90
	Ricotta cheese-whole milk	124
	Part-skim mozzarella	28
	Tomato sauce	120
	Whole wheat roll	28
	Tub margarine	5
	Fat-free milk	245
Snack	Raisins	18
	Almonds (unsalted)	28
	Day 2	
Breakfast	Tortilla, flour	49
	Scrambled egg	61
	Black beans	65
	Salsa	36
	Grapefruit	118
	Coffee	179
Lunch	Whole-grain bun	65
	Lean roast beef	57
	Part-skim mozzarella	28
	Tomato	54
	Mushrooms	39
	Corn & Canola oil	9
	Mustard	5
	Potato wedges	100
	Ketchup	17
	Fat-free milk	245
Dinner	Salmon filet	113
	Olive oil	5
	Lemon juice	10
	Cooked beet greens	38
	Corn/canola oil	9
	Quinoa	185
	Slivered almonds	14
	Fat-free milk	245
Snack	Cantaloupe balls	177
	Day 3	
Breakfast	Oat cereal ready to eat	42
	Banana	118
	Fat-free milk	123
	Whole-wheat toast	42
	Tub margarine	5
	Prune juice	256
Lunch	Rye bread	64
	Tuna	57
	Mayonnaise	14
	Chopped celery	8
	Shredded lettuce	18
	Peach	150
	Fat-free milk	245
Dinner	Cooked chicken breast	85
	Sweet potato, roasted	180
	Succotash (limas & corn)	96
	Tub margarine	9
	Whole-wheat roll	28
	Coffee	179
Snack	Dried Apricots	33
	Yogurt (Chocolate.; 0% Fat)	245
	Day 4	
Breakfast	Whole-wheat English muffin	66
	All-fruit preserves	20
	Hard-cooked egg	50
	Coffee	179
Lunch	Chunky vegetable soup + pasta	288
	White beans	101
	Saltine crackers	18
	Celery sticks	51
	Fat-free milk	245
Dinner	Macaroni pasta	57
	Cooked ground beef	57
	Corn/canola oil	9
	Tomato sauce	120
	Grated parmesan cheese	15
	Raw spinach leaves	30
	Tangerine sections	98
	Chopped walnuts	59
	Oil & vinegar dressing	21
	Coffee	179
Snack	Nonfat fruit yogurt	245
	Day 5	
Breakfast	Shredded wheat	49
	Sliced banana	75
	Fat-free milk	123
	Slice whole-wheat toast	25
	All-fruit preserves	7
	Fat-free chocolate milk	245
Lunch	Whole-wheat pita bread	57
	Roasted turkey, sliced	85
	Tomato	54
	Shredded lettuce	9
	Mustard	5
	Mayonnaise	14
	Grapes	46
	Tomato juice	243
Dinner	Broiled beef steak	113
	Mashed potatoes	140
	Cooked green beans	63
	Tub margarine	9
	Honey	7
	Whole wheat roll	28
	Frozen yogurt (chocolate)	87
	Sliced strawberries	42
	Fat-free milk	245
Snack	Frozen yogurt (chocolate)	174
	Day 6	
Breakfast	Whole wheat bread	64
	Fat-free milk	45
	Egg (in French toast)	34
	Tub margarine	9
	Pancake syrup	20
	Large grapefruit	118
	Fat-free milk	245
Lunch	Kidney beans	46
	Navy beans	26
	Black beans	36
	Tomato sauce	120
	Chopped onions	40
	Chopped Jalapeno peppers	11
	Corn/canola oil	5
	Cheese Sauce	63
	Large baked potato	299
	Cantaloupe melon	90
	Coffee	179
Dinner	Cheese pizza, thin crust	138
	Lean ham	28
	Pineapple	41
	Mushrooms	39
	Safflower oil	5
	Mixed salad greens	36
	Oil & vinegar dressing	21
	Fat-free milk	245
Snack	Hummus	45
	Whole-wheat crackers	23
	Day 7	
Breakfast	Buckwheat pancakes	146
	Pancake syrup	20
	Sliced strawberries	42
	Orange Juice (unsweetened)	248
Lunch	Canned clams	85
	Potato	170
	Chopped onion	20
	Chopped celery	15
	Evaporated milk	96
	Bacon	28
	White flour	8
	Whole-wheat crackers	46
	Orange	140
	Fat-free milk	306
Dinner	Firm tofu	114
	Chopped Chinese cabbage	38
	Sliced bamboo shoots	38
	Chopped sweet red peppers	19
	Chopped green peppers	19
	Corn/canola oil	14
	Cooked brown rice	195
	Honeydew melon	128
	Plain fat-free yogurt	123
	Coffee	179
Snack	Banana	118
	Peanut butter	32
	Non-fat fruit yogurt	245
	**DASH 7-Day 2000 Calorie Menus**	
	Day 1	
Breakfast	Bran flakes cereal	30
	Banana	118
	Low-fat milk	246
	Whole wheat bread	32
	Soft (tub) margarine	5
	Orange juice	248
Lunch	Chicken salad:	
	Chicken breast, cooked, cubed, and skinless	91
	Celery, chopped	5
	Lemon juice	3
	Onion powder	0
	Salt	0
	Mayonnaise, low-fat	9
	Whole wheat bread	32
	Dijon mustard (prepared, yellow)	15
	Cucumber slices	52
	Tomato wedges	90
	Sunflower seeds	9
	Italian dressing, low calorie	5
	Fruit cocktail, juice pack	119
Dinner	Beef, eye of the round	85
	Beef gravy, fat-free	36
	Green beans, sautéed with	125
	Canola oil	2
	Baked potato	138
	Sour cream, fat-free	12
	Grated natural cheddar cheese, reduced fat	10
	Chopped scallions	6
	Whole wheat roll:	28
	Soft (tub) margarine	5
	Apple	149
	Low-fat milk	246
Snacks	Almonds, unsalted	48
	Raisins	41
	Fruit yogurt, fat-free, no sugar added	123
	Day 2	
Breakfast	Instant oatmeal	28
	Whole wheat bagel	50
	Peanut butter	32
	Banana	118
	Low-fat milk	246
Lunch	Chicken breast, skinless	84
	Whole wheat bread	64
	Cheddar cheese, reduced fat	21
	Romaine lettuce (outer leaf)	28
	Tomato	40
	Mayonnaise, low-fat (reduced fat with olive oil)	15
	Cantaloupe chunks	160
	Apple juice	248
Dinner	Spaghetti	140
	Vegetarian spaghetti sauce	
	Olive oil	5
	Onions, chopped	12
	Garlic, chopped	2
	Zucchini, sliced	24
	Oregano, dried (ground)	1
	Basil, dried	1
	Canned tomato sauce	31
	Canned tomato paste	75
	Tomatoes, chopped	41
	Water	40
	Parmesan cheese	15
	Spinach leaves	30
	Carrots, grated	28
	Mushrooms, sliced	18
	Vinaigrette dressing	
	Garlic, separated and peeled	8
	Water	30
	Red wine vinegar	4
	Honey	1
	Virgin olive oil	3
	Black pepper	1
	Corn, cooked from frozen	83
	Canned pears, juice pack	124
Snacks	Almonds, unsalted	48
	Dried apricots	33
	Fruit yogurt, fat-free, no sugar added	245
	Day 3	
Breakfast	Bran flakes cereal	30
	Banana	118
	Low-fat milk	246
	Whole wheat bread	32
	Soft (tub) margarine	5
	Orange juice	248
Lunch	Beef, eye of round	57
	Barbeque sauce	28
	Cheddar cheese, reduced fat	56
	Hamburger bun	42
	Romaine lettuce (outer leaf)	28
	Tomato	40
	New potato salad	
	New potatoes	156
	Olive oil	5
	Green onions, chopped	4
	Black pepper	0
	Orange	131
Dinner	Cod, cooked	85
	Lemon juice	5
	Brown rice, cooked	98
	Spinach, cooked from frozen, sautéed with:	190
	Canola oil	5
	Almonds, slivered	8
	Cornbread muffin, made with oil	33
	Soft (tub) margarine	5
Snacks	Fruit yogurt, fat-free, no added sugar	245
	Sunflower seeds	9
	Graham cracker	28
	Peanut butter	32
	Day 4	
Breakfast	Whole wheat bread	32
	Soft (tub) margarine	5
	Fruit yogurt, fat-free	245
	Peach	150
	Grape juice	127
Lunch	Ham and cheese sandwich:	
	Ham, low-fat, low sodium	57
	Cheddar cheese, reduced fat	21
	Whole wheat bread	64
	Romaine lettuce (outer leaf)	28
	Tomato	40
	Mayonnaise, low-fat (reduced fat with olive oil)	15
	Carrot sticks	122
Dinner	Chicken and Spanish rice	
	Onions, chopped	32
	Green peppers	22
	Vegetable oil (sunflower)	2
	Canned tomato sauce	37
	Parsley, chopped	1
	Black pepper	1
	Garlic, minced (powder)	1
	Cooked brown rice (cooked in unsalted water)	195
	Chicken breasts, cooked, skin and bone removed, and diced	98
	Green peas, sautéed with:	160
	Canola oil	5
	Cantaloupe chunks	160
	Low-fat milk	246
Snacks	Almonds, unsalted	48
	Apple juice	248
	Dried apricots	33
	Low-fat milk	246
	Day 5	
Breakfast	Whole grain oat rings cereal	37
	Banana	118
	Low-fat milk	246
	Raisin bagel	105
	Peanut butter	32
	Orange juice	248
Lunch	Tuna salad	
	Canned tuna, water pack	34
	Celery, chopped	10
	Green onions, chopped	5
	Mayonnaise, low-fat	20
	Romaine lettuce (outer leaf)	28
	Whole wheat bread	32
	Cucumber slices (with peel)	104
	Tomato wedges	90
	Vinaigrette dressing	22
	Cottage cheese, low-fat	113
	Canned pineapple, juice pack	91
Dinner	Turkey meatloaf	
	Lean ground turkey	91
	Regular oats, dry	8
	Egg, whole	10
	Onion, dehydrated flakes	1
	Ketchup (low sodium)	12
	Baked potato	138
	Sour cream, fat-free	12
	Natural cheddar cheese, reduced fat, grated	14
	Scallion stalk, chopped	5
	Collard greens, sautéed with:	36
	Canola oil	5
	Whole wheat roll	28
	Peach	150
Snacks	Fruit yogurt, fat-free,	123
	Sunflower seeds, unsalted	17
	Day 6	
Breakfast	Low-fat granola bar	24
	Banana	118
	Fruit yogurt, fat-free, no sugar added	123
	Orange juice	248
	Low-fat milk	246
Lunch	Turkey breast	85
	Whole wheat bread	64
	Romaine lettuce (outer leaf)	28
	Tomato	40
	Mayonnaise, low-fat	10
	Dijon mustard (prepared, yellow)	15
	Steamed broccoli, cooked from frozen (boiled)	184
	Orange	131
Dinner	Spicy baked fish	
	Salmon fillet	114
	Virgin olive oil	3
	Spicy seasoning, salt-free	1
	Scallion rice	
	Cooked brown rice (cooked in unsalted water)	176
	Bouillon granules, low sodium	1
	Green onions, chopped	4
	Spinach, cooked from frozen, sautéed with:	95
	Canola oil	9
	Almonds, slivered	8
	Carrots, cooked from frozen	146
	Whole wheat roll	28
	Soft (tub) margarine	5
	Cookie	12
Snacks	Peanuts, unsalted	19
	Low-fat milk	246
	Dried apricots	33
	Day 7	
Breakfast	Whole grain oat rings cereal	37
	Banana	118
	Low-fat milk	246
	Fruit yogurt, fat-free	245
Lunch	Canned tuna, drained, rinsed	73
	Mayonnaise, low-fat (reduced fat with olive oil)	15
	Romaine lettuce (outer leaf)	28
	Tomato	40
	Whole wheat bread	64
	Apple	182
	Low-fat milk	246
Dinner	Zucchini lasagna	
	Cooked lasagna noodles, cooked in unsalted water	38
	Part-skim mozzarella cheese, grated	14
	Cottage cheese, fat-free	36
	Parmesan cheese, grated	4
	Raw zucchini, sliced	19
	Low-sodium tomato sauce (tomato and vegetable)	101
	Basil, dried	1
	Oregano, dried	1
	Onion, chopped	7
	Garlic	1
	Black pepper	0
	Fresh spinach leaves	30
	Tomato wedges	180
	Croutons, seasoned	5
	Vinaigrette dressing, reduced calorie	22
	Sunflower seeds	9
	Whole wheat roll	28
	Soft (tub) margarine	5
	Grape juice	253
Snacks	Almonds, unsalted	48
	Dried apricots	33
	Whole wheat crackers	28
